# Hypertension and primary aldosteronism diagnosis in hospitalized patients: an observation from public hospitals in Victoria, Australia

**DOI:** 10.1007/s12020-026-04562-2

**Published:** 2026-03-09

**Authors:** Stella May Gwini, Claudia Shen, Peter J Fuller, Michael Stowasser, Jun Yang

**Affiliations:** 1 Centre for Endocrinology and Reproductive Health, Hudson Institute of Medical Research, Clayton, Victoria Australia; 2 School of Public Health and Preventive Medicine, Monash University, Melbourne, Victoria Australia; 3 Disease Elimination Program, Burnet Institute, Victoria Melbourne, Australia; 4 Endocrine Hypertension Research Centre, University of Queensland Diamantina Institute, Princess Alexandra Hospital, Brisbane, Australia; 5 Department of Medicine, Monash University, Clayton, Victoria Australia

## Abstract

**Background:**

Current efforts of increasing diagnosis of primary aldosteronism (PA), a potentially curable but under-diagnosed form of hypertension, have been limited to primary care and specialist clinics, leaving out additional opportunities within healthcare. This study estimated hypertension and PA diagnosis rates during hospital admission.

**Methods:**

Public hospital admissions in Victoria, Australia, between 2007/8 and 2018/19 from patients aged ≥ 15years were retrospectively analysed to estimate rates of hypertension as principal diagnosis and PA as principal and secondary diagnosis to hypertension.

**Results:**

There were 27,749,684 admissions identified in the period, with 53.2% females and 68.2% aged ≥ 50years. Nine in 10,000 had a principal diagnosis of hypertension and 51 hypertensive patients also had PA (0.21%, 95% confidence interval 0.15%-0.26%). Principal diagnosis of PA occurred in 0.31/10,000 admissions, with higher diagnosis among males than females (0.34/10,000 vs. 0.29/10,000 admissions; p-value = 0.019) and highest amongst patients aged 50-59years (0.63/10,000 admissions) compared with 0.23/10,000 and 0.07/10,000 amongst those aged < 40years and ≥ 70years, respectively.

**Conclusions:**

Under 1% of patients admitted into hospital had a principal diagnosis of hypertension. Although this number is small, the presentations offer an opportunity for clinicians to test patients for PA. The proportion of hypertensive patients diagnosed with PA during admission was lower than the estimated 15% prevalence among hypertensive individuals, however this also highlights that PA diagnosis can occur in this setting. Future research should explore barriers and facilitators to PA diagnosis when patients are admitted to hospital with hypertension, and develop strategies to support timely diagnosis in this setting.

## Introduction

Primary aldosteronism (PA), a disorder of excess aldosterone production from the adrenal glands, is the most common endocrine cause of hypertension. PA is associated with hypokalaemia in 3% − 70% of patients [1–3]. Unlike essential hypertension which is treated by a range of non-specific antihypertensive medications, PA can be managed with targeted medication (mineralocorticoid receptor antagonists) or even cured with adrenalectomy if caused by unilateral adrenal disease. However, when left untreated, patients with PA experience a higher risk of deleterious health outcomes such as stroke, arrythmias and heart attacks than those with blood pressure-matched essential hypertension [4–7]. Early diagnosis and treatment can mitigate the harmful effects of excess aldosterone and improve health outcomes [8–10]. The newly published Endocrine Society Guidelines for PA (July 2025) recommend PA screening for all adults with a diagnosis of hypertension [11]. This highlights the importance of exploring every healthcare encounter of hypertensive patients as a potential opportunity for PA detection.

One of the current challenges for affected patients is that PA is substantially under-diagnosed internationally [12], and Australia is no exception [13]. Population-based datasets show PA prevalence as low as 0.1% [14], while prevalence studies encompassing interventions to improve screening and diagnosis rates have indicated that PA prevalence is much higher, at 12–15% in treatment-naïve hypertensive patients [1, 15] and up to 29% in people with resistant hypertension [1, 16, 17].

The detrimental impact of unmanaged hypertension is well established [18] and there is also overwhelming evidence that blood pressure control is generally poor and occurs in only 20–50% of the hypertensive population [19–24]. In a study conducted in north-west Adelaide (South Australia), close to two-thirds of subjects on hypertensive treatment had uncontrolled blood pressure [25]. The Lancet Commission on Hypertension reported that missing a secondary cause of hypertension is one of the key reasons for the lack of control [26]. Given that PA is one of the most common secondary causes of hypertension, a whole system approach to its detection and diagnosis will be instrumental in achieving better blood pressure control.

Much of the evidence on PA to date has come from general practices and specialist hypertension clinics, but many other points of care provide opportunities for diagnosis, such as specialist clinics that manage hypertension, emergency care providers, and acute hospital admissions. The aim of the current study was to estimate the proportion of patients diagnosed with hypertension or PA on admission to public hospitals across Victoria, Australia between 2007 and 2019, as well as estimate the proportion of patients with a diagnosis of hypertension during admission who were also diagnosed with PA.

## Methods

A retrospective ecological study was conducted using data obtained from the Victorian Agency for Health Information (Australia). The agency collects data from public health services around Victoria. This study included data on hospital admissions from July 2007 to June 2019, to reduce the confounding effect of the COVID-19 pandemic. Data were obtained in aggregated form by year (2007/8 to 2018/9), age group (in 5-year age bands from 0 to 4 years to 85 + years), sex (males, females and other) and local government area. The current analyses only included data from patients aged 15 years and above.

Two datasets were provided; one for hypertension as principal diagnosis (International Statistical Classification of Diseases 10th Revision (ICD-10) codes I10 and I119) and another for PA as principal diagnosis (ICD-10 codes E260 and E269). The former also included data on number of patients with an accompanying diagnosis (referred to as secondary diagnosis henceforth) of PA, hypokalaemia (ICD-10 code E876), while the latter dataset also included secondary diagnosis of hypertension, hypokalaemia and number of patients who had undergone adrenal surgery (Australian Classification of Health Interventions codes 3650002, 3650001, 3650000, 3652801, 3652800, 3652900, 9004200, 3007504). Both the principal and secondary diagnosis were as recorded on patients’ discharge summaries as conditions reflecting the patient’s episode of care during the admission.

Data were analysed using Stata Statistical Software version 18 (StataCorp. 2023. Stata Statistical Software: Release 18. College Station, TX: StataCorp LLC). Numbers diagnosed were summarised using frequencies, rates (per 10,000 admissions) and percentages. Chi-square tests and Poisson regression, with robust standard errors, were used to compare rates of diagnosis across subgroups of sex, age group and time. Poisson regression results were reported as risk ratios (RR) with 95% confidence intervals (CI). Linear trends were estimated using linear regression.

## Results

Data from almost 28 million hospital admissions were available for analyses. Approximately half (53.2%) of the patients were women and half (53.1%) aged 60 years and above (Table 1).

**Table 1:** Summary table on hospital counts with a principal diagnosis of hypertension grouped by age and sex

	Total hospital counts (*N*)	Principal diagnosis of hypertension								
		*n*	Per 10,000 admissions	Comparisons across subgroups (*p*-value)	with secondary diagnosis of primary aldosteronism			with secondary diagnosis of hypokalaemia		
					n	% of patients with hypertension diagnosis	Comparisons across subgroups (p-value)	n	% of patients with hypertension diagnosis	Comparisons across subgroups (p-value)
**All presentations**	27,749,684	24,848	9.0		51	0.2		611	2.5	
By sex *										
Male	12,983,781	7,888	6.1	< 0.001	31	0.4	< 0.001	258	3.3	< 0.001
Female	14,765,205	16,960	11.5		20	0.1		353	2.1	
**By age**										
< 40 years	5,542,046	1,810	3.3	< 0.001	7	0.4	< 0.001	75	4.1	< 0.001
40–49 years	3,269,699	2,012	6.2		8	0.4		94	4.7	
50–59 years	4,191,197	2,844	6.8		13	0.5		91	3.2	
60–69 years	5,287,794	4,142	7.8		9	0.2		111	2.7	
≥ 70 years	9,458,948	14,040	14.8		14	0.1		240	1.7	

* *n* = 698 with sex recorded as other

### Hypertension as principal diagnosis

Table 1 shows that of every 10,000 admissions, 9 patients had a principal diagnosis of hypertension, with a higher proportion among females (12% vs. 6%, RR 1.89, 95% CI 1.84–1.94) and the likelihood of hypertension diagnosis increased with age (RR 1.88, 2.08, 2.40 and 4.54 for age groups 40–49, 50–59, 60–69 and ≥ 70 years, respectively compared with those aged < 40 years; trend *p* < 0.001).

Among patients with a principal diagnosis of hypertension, 0.2% had a secondary diagnosis of PA while 2.5% had a secondary diagnosis of hypokalaemia (Table 1). A secondary diagnosis of PA was lower among females than males (RR 0.30, 95% CI 0.17–0.53) and more common among patients < 60 years old than those over 60 years (RR 3.32, 95% CI 1.91–5.76). Hypokalaemia reporting as a secondary diagnosis reduced with increasing age. More males than females had a cardiovascular secondary diagnosis (4.8% vs. 3.5%, *p* < 0.001).

**Fig. 1:**
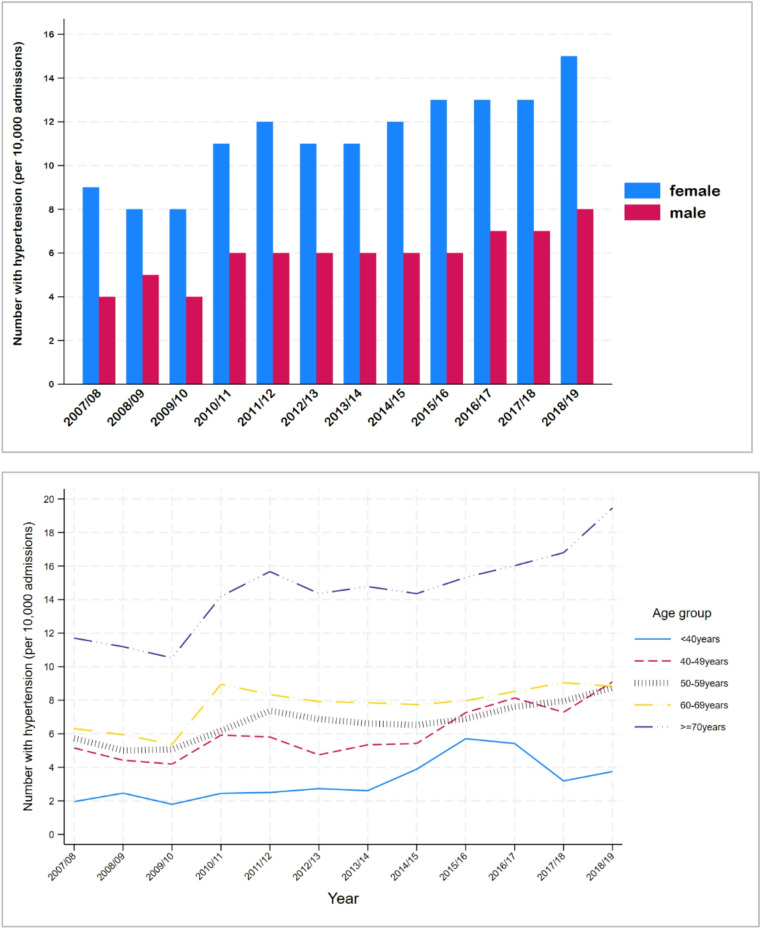
Trends in principal diagnosis of hypertension from 2007/08 to 2018/19 by sex and age group

Over the observation period, the number of patients with a principal diagnosis of hypertension increased from 6.86/10,000 admissions in 2007/08 to 11.73/10,000 admissions in 2018/19 (increase of 0.43/10,000 per year, p-value for trend < 0.001). Figure 1 shows that the proportion of both males and females with a principal diagnosis of hypertension increased over the observed time period (RR 1.048, 95% CI 1.041–1.055; RR 1.052, 95% CI 1.056 per year males and females, respectively).

The trend in hypertension diagnosis also varied by age-group, with increases of 0.25, 0.36, 0.27, 0.25 and 0.62 per 10,000 admissions per year among those aged < 40, 40–49, 50–59, 60–69, ≥ 70 years respectively (comparison of trends *p* = 0.023). Only the trend for those aged ≥ 70 years significantly differed from the youngest age group (*p* = 0.005).

### Primary aldosteronism as a principal diagnosis

Of the observed hospital admissions, 869 (0.31/10,000 admissions) had a principal diagnosis of PA, with a significantly higher rate among men than women (Table 2). The rate of PA diagnosis increased with age, peaking among those aged 50–59 years. Just over 10% of patients with a principal diagnosis of PA had a secondary diagnosis of hypokalaemia and a fifth had adrenal surgery.

**Table 2:** Summary table on hospital admission counts with a principal diagnosis of primary aldosteronism, grouped by age and sex

	Total hospital counts	Principal diagnosis of primary aldosteronism								
		*n*	Per 10,000 admissions	Comparisons across subgroups (*p*-value)	with secondary diagnosis of hypokalaemia			Undergoing adrenal surgery		
				N	n	% of patients with PA	Comparisons across subgroups (p-value)	n	%	Comparisons across subgroups (p-value)
**All presentations**	27,749,684	869	0.31		111	12.8		176	20.3	
By sex*										
Male	12,983,781	441	0.34	0.019	56	12.7	0.95	100	22.7	0.07
Female	14,765,205	428	0.29		55	12.9		76	17.8	
**By age**										
< 40 years	5,542,046	127	0.23	< 0.001	17	13.4	0.88	19	15.0	0.49
40–49 years	3,269,699	180	0.55		24	13.3		36	20.0	
50–59 years	4,191,197	265	0.63		29	10.9		59	22.3	
60–69 years	5,287,794	235	0.44		32	13.6		51	21.7	
≥ 70 years	9,458,948	62	0.07		9	14.5		11	17.7	

* *n* = 698 with sex recorded as other

The rates of PA diagnosis increased over time by 0.039/10,000 admissions per year (*p* < 0.001 for trend). Overall, the main changes in PA diagnosis commenced after 2012/2013, with a very small change of 0.0007/10,000 admissions before 2013/2014 (*p* = 0.937 for trend) and a greater change of 0.069/10,000 admissions per year thereafter (*p* < 0.001 for trend).

**Fig. 2:**
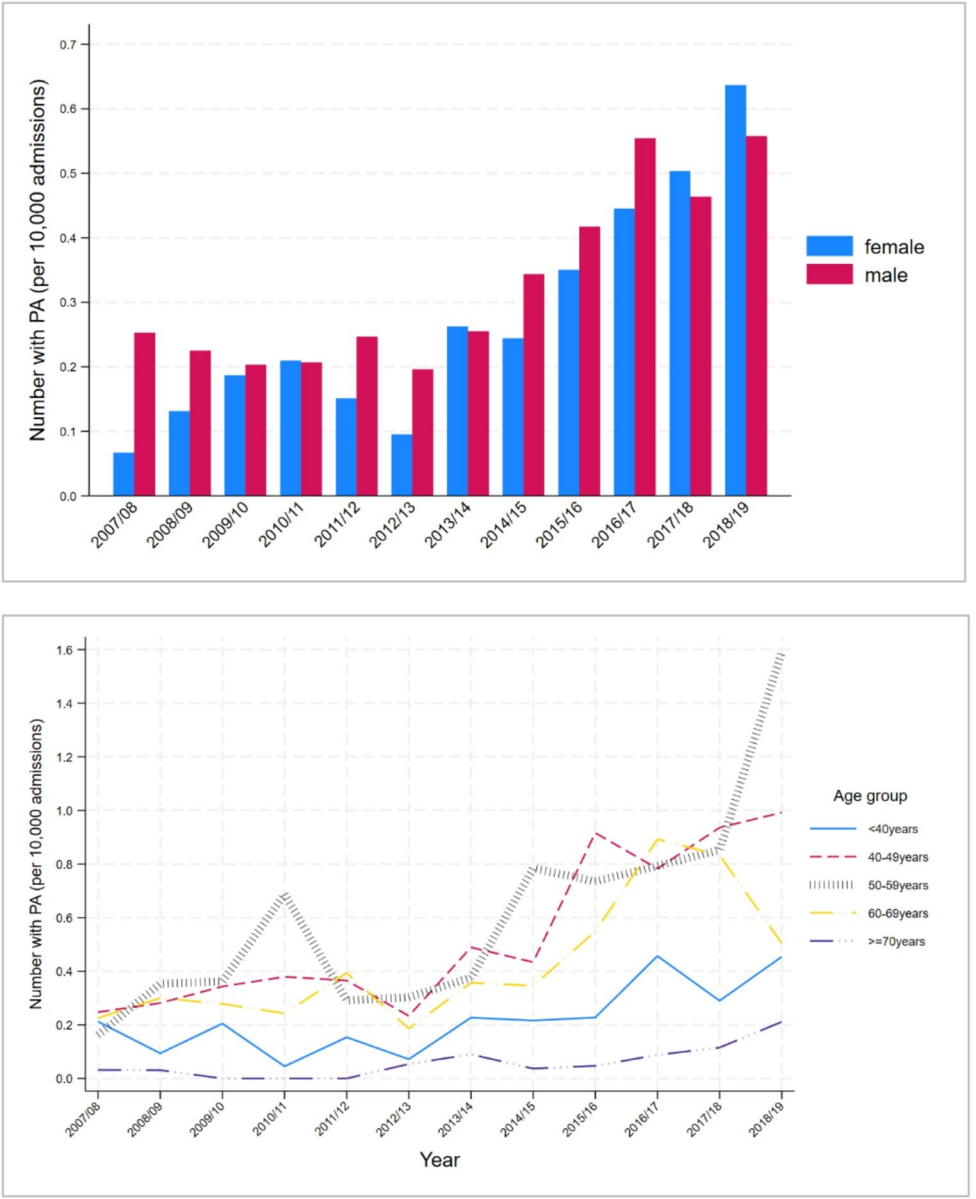
Trends in principal diagnosis of primary aldosteronism by sex and age grou

Figure 2 shows the rates for principal diagnosis of PA for each year by sex and age group. In the period after 2012/13, the increase in principal diagnosis of PA differed across age groups (*p* < 0.001 for comparison of trends), with small changes in the < 40 years and ≥ 70 years age groups (0.026/10,000/year, *p* = 0.013 in < 40 age group and 0.013/year, *p* = 0.012 in ≥ 70 age group). The largest changes after 2012/13 were in the 40–49 (0.129/10,000/year, *p* < 0.001), 50–59 (0.172/10,000/year, *p* = 0.001) and 60–69 age groups (0.081/10,000/year, *p* = 0.044).

## Discussion

The analysis of hospital admissions data over a 12-year period revealed an increase over time in the number of patients with a principal diagnosis of hypertension or PA. When contrasting earlier versus recent years, it is encouraging to observe more patients with a principal diagnosis of hypertension also had a secondary diagnosis of PA. However, the proportion of patients with a principal or secondary diagnosis of PA is still lower than expected. There were also more patients with hypertension and hypokalaemia than there were hypertensive patients with PA. Rates of hypertension and PA diagnosis varied by sex and age.

There is scarce information on the prevalence of hypertension in hospital admissions. In a Brazilian study, 0.73% (i.e. 73 per 10,000) of hospital admissions were attributed to hypertension, with higher rates observed among admitted women [27]. An earlier systematic review published in 2011 showed varying proportions of hospital patients diagnosed with hypertension [28], with 17% reported in the most recent included study of patients aged over 65 years [29]. In an America study published in 2022, 1% of patients admitted into hospital were admitted for hypertension [30]. Despite all these statistics being much higher than the prevalence of 9 per 10,000 observed in our study, the substantial differences in health systems (e.g. universal healthcare funding in Australia vs. mixed funding in the United States of America; varied use of primary and tertiary healthcare services for hypertension diagnosis and management) and included cohorts between these countries and Australia render the data difficult to compare. Additionally, the research context and populations covered by these studies vary. For example, the study by Ghazi et al. [30] only considered prevalence of severe hypertension in patients admitted for 2–30 days in specific hospital wards while our current study is using a more population based approach. Additionally, it is unclear from the previous studies whether the reported rates were for principal diagnosis of hypertension alone, or they also included secondary diagnosis. In general, though, hypertension is an outpatient disease, so only those with severe disease would require hospital admission, which makes it even more likely that a secondary cause such as PA may be responsible.

The rate of hypertensive patients subsequently diagnosed with PA in our study was much lower than expected. Similar to hypertension diagnoses, there is little literature specifically reporting on the number of patients with a PA diagnosis on admission to hospital. On the other hand, data from specialist clinics (including day clinics and overnight admissions) suggested a PA prevalence as high as 35% [1, 16, 17, 31, 32], much higher than the 0.2% observed in this study. Worth noting is that more patients among those with a principal diagnosis of hypertension had hypokalaemia (2.5%) than had PA (0.2%) even though the combination of hypertension and hypokalaemia is a hallmark of PA. Moreover, about a quarter of people with concurrent hypertension and hypokalaemia may have PA [2]. Due to the nature of the data used for this study, it is not possible to ascertain whether those with hypokalaemia were also screened for PA, nor is it possible to ascertain whether patients without a record of hypokalaemia or PA were screened negative. However, not all persons with PA have hypokalaemia and in the current study, only 13% of those with a principal diagnosis of PA also had hypokalaemia. Thus, even if all the people admitted with hypertension and hypokalaemia were tested for PA, there would still be a larger cohort with normokalaemia who should be tested.

It can be acknowledged that complete PA diagnosis in hospital may not be possible and the process of PA diagnosis, from screening to diagnosis and subtyping, is complicated and long. Some of the factors that alter the renin-aldosterone axis may make it difficult to interpret inpatient ARR results, unless clearly abnormal. Such factors include intravenous saline administration, acute illness, physiological stress with elevated cortisol/ACTH, changes in blood volume status and electrolytes and reduced mobility. Use of current PA/screening guidelines which recommend screening in ambulatory patients, seated after at least two hours upright in the morning, can help minimise these confounders. A pragmatic approach, however, is to flag high-risk individuals during admission and arranging testing once they are clinically stable in an outpatient setting, ideally soon after discharge. This strategy supports timely identification of PA while maintaining adherence to guideline-based testing conditions and optimising diagnostic accuracy.

The current study also showed that since 2012/13, the rate of PA diagnosis in Victorian hospitals increased. This may reflect the growth in research on PA internationally [33], with a consequent increase in the awareness of PA. The establishment of an Endocrine Hypertension Clinic providing dedicated care for patients with PA at one of the major Victorian teaching hospitals may have specifically impacted hospital admissions [34, 35]. The discrepancy, though, between known PA prevalence in high risk populations and the diagnosis rates in this study render a need for improved detection and diagnostic strategies.

## Strengths and limitations

To the best of our knowledge, this is the first study to examine PA screening in hospital admissions based on a population-based dataset. This provides more robust estimates than previous smaller cohort studies, which have typically focused on selected sub-populations and relied on survey data prone to recall and other biases. Nevertheless, the data used in this study were collected for clinical management, reporting and administrative purposes, not research, which limits the breadth of analyses that could be conducted. While patient discharge summaries generally record new diagnosis as principal and secondary diagnoses, it is possible that hypertension or PA that were existing prior to admission were subsequently recorded whilst the patient was in hospital. Due to the nature of the data, granular detail on the nature of the diagnosis, whether PA screening was conducted or not, how the diagnosis of PA was made and whether it is new or existing were unavailable for analyses. However, even if all diagnosis were considered as new in-hospital diagnosis, the rate of diagnosis amongst hypertensive patients was still very low compared to the estimated prevalence.

The risk of misclassification bias is also likely in these data. In particular, coding of secondary diagnoses may not be as accurate as for principal diagnoses [36, 37], hence the proportion with a secondary diagnosis of PA may be under-reported. Additionally, hypertension is more often coded as a secondary cause for admission, rather than principal diagnosis. Hence, it is likely that this study underestimates the number of missed opportunities for PA screening. Another limitation of our study is that only confirmed diagnoses were captured; pathology data were unavailable to determine whether patients were tested for hypokalaemia and/or PA but returned negative results.

## Conclusions

Data from hospital admissions in Victoria revealed that PA remains an under-diagnosed cause of hypertension, even among patients with concurrent hypertension and hypokalaemia. The increasing prevalence of PA in hospital admissions over time is promising but falls short of the expected prevalence. Proactive evaluation for PA in patients admitted with hypertension as their principal diagnoses may be an important strategy to improve PA detection, in accordance with PA guideline recommendations and with considerations of factors limiting accurate interpretation of ARR. Shih et al. (2024) [38] provides actionable recommendations for hospital doctors to consider when encountering hypertensive patients, and following these recommendations would help break barriers to effective screening of primary aldosteronism.

## Data Availability

No datasets were generated or analysed during the current study.
